# Selections and Abstracts

**Published:** 1900-10

**Authors:** 


### SELECTIONS AND ABSTRACTS.

### The Conservative Tendency in Genito-Urinary Work.

In defining the position of conservatism, I shall adhere to the accepted definition, "the disposition and tendency to preserve what is established," as opposed to radicalism, "the resorting to extreme measures—to go to the very root and eliminate all other considerations."

This gives a latitude to the subject in hand not apparent at first glance. It is a popular fallacy that the conservative is he who hangs back, objecting to every advance. On the contrary, he is found at the head of every permanent movement, only demanding, first, that proof be shown of the wisdom of such movement; and so it may be insisted that every substantial, permanent advance has conservatism for its mainstay. The conservative course is almost always the safe course, while very often the radical measure is fraught with many dangers.

It is my purpose to touch on only a few points in connection with genito-urinary work, where the wisdom of such conservatism has been made manifest.

Gonorrhea, a disease of such dreadful consequence as to have led many men most familiar with it to consider it even more of a menace to society than syphilis; a disease which Lawson Tait averred every man to have suffered from at least once in his life (this statement I utterly discredit, and venture the opinion that not 50 per cent. of men are so unfortunate); a disease which has, I suspect, been the greatest single factor in the development of gynecology; a disease whose awful significance is even yet, at the threshold of the twentieth century, improperly understood or indifferently considered by the majority of practitioners. All during the earlier centuries, and even up to recent times, it was considered as a sort of joke by profession and laity alike, and even now the likening of it to a bad cold is a daily experience.

The various treatments have been too numerous to attempt mention, and the very fact of the many so-called specific treatments and patent sure-cures is proof positive that none of them fulfil the claims made. Radicalism once taught the "abortive treatment," a measure we now know to be a dreamy impracticability.

I quote the direction for such treatment, as given me by one of the most famous of the older teachers in one of our great medical schools:

1. Abortive treatment must be undertaken very early, before discharge becomes profuse or purulent in character.

2. Compress urethra after urination just behind peno-scrotal juncture.

3. Anterior to this freely irrigate with $AgNO_3$, 1-500. Sufficient power is acquired by using a fountain syringe at the height of six feet. The whole glans is thoroughly cleansed with this same solution. This is repeated to thoroughly distend urethra.

4. Now by use of hand syringe distend with five-per-cent. solution of $AgNO_3$ and retain three minutes.

5. Now complete with repetition of 1-500 strength, and apply sterile cotton.

There is the strongest probability that if ever this apparently cured an urethritis it was of the simple form, without the gonococcus being present, and would have subsided in a few days, independently of treatment. At the present time we have the host of organic forms of silver said to cause complete disappearance of gonococcus after a very few applications.

Janet, with his fourteen irrigations and the innumerable modifications, all these are to be classed among the radicalisms of treatment. They do not do anything like what is claimed, and they fail because of the fact that so little cognizance is taken of the pathological conditions present and constitutional traits back of all.

These enthusiastic knights, who charge forth impetuously in the crusade against all who claim a reasonable time to cure a gonorrhea, are, as a rule, totally forgetful of the forces they have to deal with. They seem to fancy that these microbic forms are standing tip-toe on the surfaces of cells, reaching out into the open caliber of the urethra, only waiting to be figuratively knocked off with a long pole and done away with.

Bumm taught that the gonococcus made its way down between the epithelial cells toward the sub-epithelial connective tissue layer, and then, and not until then, did the reaction of the tissues occur and first subjective symptoms appear. We know, moreover, that the cure is due to a process akin to cicitrization, the throwing forward of a new pavement of epithelium, which advances as a resisting wall, ultimately literally crowding out all morbific elements. And so in the light of pathologic process and tissue-change, coupled with the clinical experience that takes into consideration different degrees of resistance offered in different cases, differing degrees of virulence of infection, personal idiosyncrasy, etc., the conservative workers still consider a "clap" cured in four to six weeks as well done, and make no positive promise in any case to be able to repeat such a success. There has been so much charlatanism in connection with this subject that we are often, perhaps, too prone to attribute dishonesty to such claimants.

I apprehend that some, in their wild claims of treatment, are simply mistaken, and blinded by over-zealousness and hopefulness for what has seemed remarkable in a limited number of perhaps specially selected cases. Irrigation properly applied has wonderfully beneficial effects; protargol, mercurol, and similar salts are of great value. Still there remains the fact that each case is its own law, and the universal specific has not yet appeared. Again, many a chronic discharge or gleet can be traced to too vigorous measures, to over-treatment.

If the microscope repeatedly shows a discharge or moisture to be made up of mucus and epithelium, and free from specific elements, the wisest course, the conservative course, is to cease all local tamperings and give nature a chance to overcome the catarrhal condition that is to be expected after an acute inflammation of any mucous membrane.

Let me cite a typical case, a young man with profuse gleety discharge, diagnosed by several well-known and careful men as granular urethritis, anterior and posterior. For more than a year he had gone from one to the other, and each in his turn, anxious to accomplish something, had persisted in local applications. He had practically for one year been sounded every five days to 32 F., followed by instillation of one grain to one ounce of nitrate of silver. He manifested pronounced hypochondriasis.

Is it any wonder that his granular patches and discharge persisted? Under a do-nothing policy, so far as local treatment was concerned, along with tonics, he was to all intents and purposes well in one month from the time I first saw him.

Dr. Otis, in his distinctly radical stand as to urethral calibers, led to a great stride forward. He, however, in his zeal overstepped the mark, and went far to the wrong. The pendulum finally swung back to safe anatomically-established grounds, Dr. Otis himself modifying his first views, and to-day we conservatively regard the urethra as a canal, with peculiarities all its own and peculiarities to be respected. Instead of going literally "fire and sword," and slashing every meatus up to such a point that a man's urine would drop out without either force or direction between his feet, we have devised dilators to reach that particular part requiring dilatation, and, instead of cutting vast numbers of strictures of large caliber, we now recognize them as points of normal narrowing, and keep meddling hands off. We would not praise Dr. Otis any the less for what he has done, but simply insist that arbitrary rules without proof should be considered very cautiously, if at all.

In the consideration of uro-genital tuberculosis we pause and consider the insidious and far-reaching process of tubercular change, and find that a few years ago, where operative measures were rushed into in the hope of complete eradication of local change, the surgeon of to-day withholds his hand and operates little, hoping by sunlight, good food, and proper general medication and hygiène to enable the natural constitutional resistance to assert itself, accomplishing in this way often what cannot be hoped for from operative procedure.

No more fatal blunder could be made than in case of tubercular base of bladder to resort to indiscriminate instrumentation for mistaken purposes of exploration or treatment. As soon as such condition is ascertained, intelligent conservatism dictates that we refrain from any foolish course having in view vigorous local specific treatment.

On February 11, 1880, Henry Morris, that most eminent English surgeon, "successfully removed a mulberry calculus weighing thirty-one grains from the undistended and, to the naked eye, quite normal kidney." Speaking of this case, he says: "It demonstrated for the first time that a stone could be removed safely by cutting freely upon it through a thick layer of renal tissue. It thus became the starting-point of both the development and the conservatism of renal surgery, which, I am persuaded, will become more and more conservative in the future."

He points out that the old expectant treatment of renal calculus is obsolete, dangerous, and unjustifiable. If renal calculus is present, it should be by operative measure removed, regardless of immediate symptoms. It will be the means of saving many a kidney, and the old saculated and degenerated so-called "surgical kidney" will have become a far greater rarity than it is at present. Again, Morris says: "So useless is medicinal and expectant treatment that I have refused to attend consultations in cases of calculous anuria unless I have permission beforehand to operate at once if I think the case suitable."

The object of this paper has been an attempt to show briefly the conservative tendency as we become better observers of anatomical, pathological, and clinical conditions. Every theory, doctrine, or drug should be generally kept at arms' length until it has been tried by these measures and found good. The shelves of pharmacists and libraries of doctors would not then be filled with such masses of rubbish as now encumber them, and there would likewise be fewer unfortunates going about our land helpless and hopeless victims of some butchering orificial surgeon, or similar impractical and theorizing visionary.—*F. R. Charlton, M.D., in American Practitioner and News.*

---

### THE TONGUE AS AN INDEX IN DISEASE.

I may not be able to bring out anything new on this subject, not because I have not looked through all the journals and standard works at my command, but on account of the absence of anything new being mentioned by our most recent authorities, except as being mentioned occasionally in the description of some condition on disease.

It is needless for me to show you the importance of this subject, because there is no room for doubt in the minds of all practitioners of medicine as to the importance of the appearance of the tongue in disease, taken in connection with other symptoms, and that its inspection is part of the routine of every medical examination. The appearance of the tongue is, to a certain extent, well known, even to the laity. We are not so apt to mistake the appearance of the tongue in diseases with which we daily meet, but then there are diseased conditions occasionally met with which are accounted for by some change in the tongue, which we are apt to overlook. And the presence or absence of disease in other parts is established by the changes in sensibility, form, color, mobility, etc.

Again, too much reliance must not be placed on the signs furnished by the tongue alone, for you might fall into error on account of the fact that its appearance is modified by so many and so slight causes. In examining the tongue we have to note its form and volume, the degree of moisture, the condition of its surface, whether smooth, fissured, ulcerated, or the seat of an eruption; its color, coating, movements and its tactile and gustatory sensibility.

It is well to note the appearance of the tongue in health. The tongue in health varies greatly, but is usually of a rosy red color, moist, covered with minute papillæ, free in its movements and almost constantly changing in form. Many persons have a coated tongue in health, especially smokers, tea-tasters, etc. As to volume, in some it is narrow and pointed at its tip; in others it is large and flabby, with thick and rounded tip and margins often marked by the teeth. In the nervous individual the tongue is quickly protruded and quickly withdrawn. In the phlegmatic the motions of the tongue are sluggish.

In old age the tongue is almost always coated, and is less moist than in younger years, becoming very dry and glazed even at the outset of the slightest ailment; and this fact is of importance, as we may be led to consider a case as serious which is only suffering with a slight ailment, if we consider the evidence furnished by the state of the tongue alone. The tongue is not changed in every abnormal state, and frequently presents no change in cancer of the stomach, or in gastralgia, or enteralgia. In simple jaundice, nasal catarrh and many other slight ailments unaccompanied by fever, the tongue often presents the normal appearance.

Before taking up the appearance of the tongue in the different individual fevers, I will mention briefly the characteristics of tongue in different diseases and affections as to its form, volume, degree of moisture, surface, color, coating, movements and sensibility.

First, as to form. Owing to the absence of moisture the shape of the tongue, in many cases, is changed, and is frequently found concave on its upper surface from contraction of the dried epithelial layer. According to Gubler, there is contraction of the transverse muscular fibers, so that the organ is less broad than normal.

In hemiplegia the tongue is altered in consequence of the unilateral paralysis, and we find the affected side soft and flat, and after a time may be reduced in volume.

Hemiatrophy of the tongue has been observed by Ballet in a number of cases of locomotor ataxia. There is a noticeable diminution in the size of the organ, and its surface is drawn up in ridges. This, according to Ballet, is of great importance, and may be present before any of the manifestations of the disease present themselves.

As to size. When there is interference with the return circulation, as in heart disease, pulmonary affections, or compression from any cause of the veins of the neck, the tongue is often swollen. In chronic diseases of the chylopoietic system the organ is often enlarged, and shows on its margins the indentations from pressure against the teeth. In idiots and others suffering from mental disturbance, the tongue is often large and flabby.

In general paralysis, paraplegia, and sometimes in hemiplegia the tongue is large, soft and flabby, its dimensions being such as sometimes to prevent closure of the mouth.

In anemia the tongue is broad and flabby, and shows the marks of the teeth. This condition is also found in smallpox, typhus fever, scurvy, and various blood discrasia and in mercurial poisoning.

Inflammation with turgescence of the organ occurs as a result of the local action of certain irritant and corrosive poisons swallowed accidentally or with suicidal intent.

Atrophy of the organ not infrequently occurs with similar changes in other muscles in the course of progressive atrophy.

As to moisture. In ptyalism there is an increase in the lingual as well as of the salivary glands. A return of moisture is a favorable sign in adynamic conditions. Dryness in a slight degree is present in some persons who are enjoying good health. It is present in obstruction of the nasal passages, dyspnea (that is, occurring with phthisis, heart disease, pneumonia, etc.), mouth breathers, certain drugs as belladonna, speakers, long-continued fasting, prolonged febrile conditions, especially when the temperature remains high, inflammation of the abdominal viscera, intestinal obstruction, etc.

As to surface. The surface of the tongue in health is convex on its upper surface, and somewhat slightly roughened from projection of papillæ. When moderately dry it is smooth and presents a glazed appearance from the presence of inspissated mucus.

Fissures of the tongue occurring without furring, or dryness, are found in gastric disturbances, but may also be present in some even in health.

Mucous patches, gummy tumors, etc., occurring in syphilis may also present fissures. Ulceration of the frenum are sometimes present in children suffering from whooping cough.

Wounds caused by teeth in epilepsy are of importance, especially where the convulsions are nocturnal.

As to color. In anemia, after profuse hemorrhages and in wasting diseases, the tongue is pale. In icterus, Addison's disease, etc., the color of the tongue approaches that of the skin.

It is abnormally red in scarlatina, inflammatory fevers and often in the beginning of smallpox.

A red moisture indicates debility, as where it is caused by long-continued, exhausting discharges. In chronic conditions affecting permanently, in a measure, the digestive organs, the tongue is often intensely red. In all gastric disturbances the tongue is usually redder than normal, and is sometimes of a brown red color. It is sometimes of a purplish venous hue, where there is some impediment to the circulation, or where there is an interference with hematosis, and we see this color in many cases of heart disease, in cholera, in croup, in pulmonary affections, causing asphyxia, and when large effusions are present in the pleural and abdominal cavities. In malarial fevers the tongue is often purplish at its margins and in plethoric conditions it is also found present.

A black tongue is observed in certain cases, but its diagnostic significance has not been determined.

The tongue is often discolored by various alimentary substances, drugs or corrosive acids, such as blackberries, tobacco, rhubarb, etc. Sulphuric acid, when swallowed in a concentrated form, blackens the tongue and blackens the buccal mucous membranes; nitric or cromic acid causes a yellow or blackish stain, according to depth of eschar. Carbolic acid whitens the parts. The acid nitrate of mercury produces a gray and gelatinous appearance; argentic nitrate gives a white or pearl-gray eschar.

DaCosta, quoting Chambers, says, that tea-tasters have often a smooth, orange-tinted coating on the tongue.

As to coating. This condition is even noticeable to the laity, and, in fact, is almost always indicative of some departure from the normal. There are a few exceptions where the tongue is furred in health and a thin, whitish coating is always seen in smokers. When normal secretion is interfered with, as in fevers, there is less chance for the detached epithelium to be washed away, and the coating is further increased by the collection of inspissated mucus. The coating and discoloration taken together, furnish a valuable diagnostic symptom. A white coating indicates febrile disturbance or slight indigestion; a yellowish tinge points to hepatic disturbance; and a dry, brown fur, except in mouth-breathers, is usually a sign of profound discrasia. The fur may be moist or dry, the former in gastric disturbances and the latter in prolonged febrile disturbances.

A sign of some importance in diagnosis of gastric ulcer from cancer is the presence of a coated tongue in the first named and the absence in the latter unless there is fever.

A hemorrhagic coating results from the inhibition of the coloring matter of the blood. A yellowish fur forms on one side of the tongue as a result of the irritation caused by carious teeth. In certain cerebral affections, especially in apoplexy, the tongue is covered with a thick, sticky fur, and it is also coated in chronic alcoholism.

As to movements. The movement of the tongue should be studied, and any abnormal affection of speech, or deglutition arising from defect or disorder of these movements should be noticed. They should be studied at rest, and then it should be moved from side to side, or in different directions, and then the patient should be directed to protrude the tongue and make similar movements. This mode of investigation gives, in certain cases, important information. The movements of the tongue are sluggish in all adynamic conditions, in mental hebetude and when organ is dry. In paralysis of various kinds the muscles of the tongue are often affected and tongue cannot be protruded. In paraplegia one side of the tongue may be affected, and when protruded it deviates toward the paralyzed side, being pushed that way by the sound muscles. In adynamic conditions, in alcoholism, lead poisoning, paralysis agitans, and when the individual is under the influence of various emotions, such as fear, anger, etc., a trembling of tongue is observed.

In progressive muscular atrophy, in general paralysis of the insane and in locomotor ataxia, fibrillary contractions are sometimes observed.

Convulsions, involving the lingual muscles, have been observed in hysteria, epilepsy, chorea and in tubercular meningitis in infants.

As to sensibility. In paralysis tactile sensation is often lost, but this is not of much importance, as the affection is usually determined by other symptoms. Disorders of taste are common in hysteria, and are also present in early pregnancy.

In dyspepsia and catarrhal conditions of the stomach patients often complain of a bad taste in the mouth, especially on rising in the morning. In some cases there is a loss or a perversion of taste, as in diabetes mellitus there is a mawkish taste in the mouth, and all substances seem sweetened.

The principal conditions and diseases in which the tongue affords useful information, especially those not already mentioned, are as follows:

1. Nervous Diseases:

These have already, to some extent, been mentioned, but I may also add that it is of importance to observe the manner in which a patient protrudes his tongue, or attempts, when asked to do so, as this may be made use of to indicate the state of consciousness in various conditions affecting the brain.

In many cases of cerebral disease the tongue shows a marked and speedy tendency to become thickly furred and very foul. The jerking and irregular movements of the tongue are well marked in many cases of chorea. In cases of severe neuralgia of one side of the face, the tongue occasionally presents peculiar appearances, as unilateral furring, thickening of the mucous membrane or enlargement of the papillæ. During attacks of migraine its surface usually becomes much furred.

2. General Conditions:

In the febrile state, as has already been briefly mentioned, the tongue is altered, whether associated with specific fevers or with inflammatory diseases. It becomes covered with more or less fur, often of considerable thickness, and usually white or yellowish white.

The conditions found in particular fevers will be dwelt on to a fuller extent later on, in considering each one of the most important separately.

In the "typhoid state," whatever this condition may be associated with, the tongue is dry, and usually covered with a brown or even a blackish crust, appearing as if it were baked. The tongue also indicates the general tone of the system, being usually large and flabby when this is below par. In some instances of diseases attended with marked general wasting, the tongue comes to present an irritable appearance.

3. Conditions Affecting Blood and Circulation:

In anemia it is more or less pale; generally large, flat and flabby; and frequently at the sides of the teeth. In the plethoric condition it is also large, but tends to be a deeper color than normal and may present a venous tint. The anemic tongue may be quite clean. A temporarily congested condition of the digestive organs and liver is supposed to produce a corresponding plethoric state of the tongue. Any cause of general obstruction to the venous circulation, from whatever cause, is likely to give rise to enlargement of the tongue, and to make it assume a congested, or even a cyanotic appearance, in marked cases, such as those of congenital malformation of the heart.

4. Affections of the Alimentary Canal and its Related Organs:

The tongue is peculiarly liable to be altered in affections of the mouth and throat. In catarrh of these parts it is more or less furred. In tonsillitis there is usually a very thick fur; and it may be most marked on the side of the inflamed tonsil. Even a local irritation, such as that caused by decayed teeth, may originate a fur, but in such cases it is usually localized.

In any acute disorder of the alimentary canal the tongue speedily becomes furred, usually white or yellowish white, but it may be more or less brown, as in so-called acute dyspepsia, catarrh of the alimentary canal and hepatic disorders. In severe acute gastritis the organ often presents a strikingly red and irritable appearance, especially at the tip and edges, with enlarged papillæ and a tendency to dryness. In chronic forms of dyspepsia and gastric catarrh, the tongue presents different appearances. In the atonic variety it is usually large, flat, soft and flabby; frequently marked with the teeth, more or less furred, though it may be quite clean. In the irritative form it tends to be small, elongated and pointed, contracted and firm; red and irritable; with elongated papillæ; and generally only having a thin white fur, through which these papillæ project, but it may appear unusually clean and raw looking. In the more grave diseases of the stomach, namely, cancer and ulceration, the tongue may be quite clean and healthy looking.

In the affections of the intestines, not complicated with a deranged stomach, there is no change in the appearance of the tongue.

Constipation, especially if habitual, tends to make the tongue large and furred, particularly if associated with portal congestion and deficiency of bile. In some cases of chronic intestinal catarrh, with diarrhea, the organ presents an irritable appearance. In chronic dysentery it often becomes red, glazed or fissured.

Although I have already mentioned the appearance of the tongue in a number of diseases under the headings: Sensibility, form, color, mobility, etc., as well as under nervous diseases, general conditions, conditions affecting the blood and circulation, and those affections of the alimentary canal and its related organs, yet I will run the risk of repeating some things already mentioned, but not brought out fully, as to the appearance of the tongue in a few of the more common diseases, and briefly mention the appearance in a few special diseases.

Typhoid Fever.—Flint says "the tongue may be simply furred or frosted, but it is oftener covered with a coating more or less thick, which, in different cases, is whitish, brownish or even black. Not infrequently the coatings are thrown off once or repeatedly, and the surface is thin, usually reddened.

"Exfoliation of the coating, or its gradual thinning, the surface being moist and of a natural color, betokens convalescense. The varied appearance of the coatings have no special significance. The surface of the tongue is sometimes reddened and smooth or glazed. It sometimes becomes dry and hard. This occurs in coma vigil. The patient breathing with the mouth open, the surface is dessicated. It sometimes becomes cracked and deeply fissured.

"Tremulousness of the tongue, as in cases of delirium tremens, is sometimes observed, usually preceding or accompanying grave ataxic symptoms. The tongue, in some cases, at an advanced period, is protruded with apparent hesitation and difficulty, and when protruded may not be withdrawn, apparently from forgetfulness. The delay in withdrawing and protruding the tongue represents the weakness and slowness of the mental acts."

The red glazed tongue, as given by different authorities as one of the diagnostic symptoms of typhoid fever, is rather deceiving. In fact, you have all observed cases, especially early in the disease, with nothing but a whitish coating, and in some cases not any coating to speak of. In most cases of typhoid fever the tongue is small and irritable, with enlarged papillæ and a thin whitish or yellow fur; or sometimes it is red, smooth and glazed or shining.

Typhus Fever.—The tongue, more frequently than in typhoid fever, becomes covered with a thick brown or black coating. It is less frequently reddened, glazed and fissured.

Erysipelas.—The tongue is covered with a thick, dry coat, which may even be crusty.

Scarlet Fever.—In scarlet fever the papillæ tend to become much enlarged and prominent, so that they project through the fur, and the tongue presents the so-called strawberry appearance. This is also described as if cayenne pepper or red sand had been sprinkled over it. This strawberry tongue is not the first diagnostic symptom of scarlet fever, but usually comes on in a few days after the initial vomiting, and is well marked after the mild diarrhea, which is usually present early in the disease. The tongue is slightly coated even at the outset.

Trousseau gives the following description of the appearance of the tongue from the first day. He says the appearance of the tongue is a specific as are the pustules on the mucous membrane of the mouth in smallpox. On the first day there is only a slimy fur, more or less thick, more or less white, and which, if the patient has vomited, has a yellow or green color; at the point and edges there is only a slight redness. On the second day the redness increases in intensity and extent, and this change continues to the third day. About the fourth or fifth day the saburral coating has almost or altogether disappeared; the whole tongue is then scarlet and swollen and the papillæ rise above the level of its surface in such a way as to give it a strawberry-like aspect.

Dengue.—Dengue, in a few hours after its appearance, presents the following: The tongue is covered with dense white paste, or with a thick, dirty white coating, always moist and associated with a disagreeable bitter taste in the mouth.

Relapsing Fever.—The tongue presents a white or a yellowish-white fur, while the following peculiar condition has been noted: A small triangular space, towards the point of the tongue, as well as at its edges, is clean and often redder than normal. In mild cases the tongue continues moist throughout the whole attack; but in more severe, dryness, blackness and incrustation with sordes occur.

Measles.—The tongue, thickly coated, is mostly moist and few red papillæ may be often observed to project through the thick coating of fur.

Diphtheria.—Tongue is moist, with a thin, creamy fur, and the diphtheritic deposit may be deposited on its surface.

Cerebro-Spinal Fever.—The tongue is frequently large and flabby, showing indentations made by pressure of the teeth. Reynolds says: The tongue in this disease is as frequently clean and moist as dry, foul and discolored. And the changes in the tongue are usually due not to the disease itself, but depend on the degree of febrile excitement or the development of the typhoid state.

Yellow Fever.—The tongue is moist and more or less coated.

Influenza.—The tongue is usually moist and covered with a white creamy fur; but occasionally it is morbidly red at the tip and edges and thickly coated with a white brown fur towards the center and root; more rarely, and especially in the morning, it is dry.

Parotiditis.—The tongue is furred, but usually moist.

Rheumatism.—There is generally a very thick, creamy fur, as well as preceding and during attacks of gout, and in the latter especially it often becomes brownish.

Mild Dysentery.—The tongue is white and moist.

Acute Dysentery.—The tongue is foul in the center, becomes red at the edges and dry, then dark brown and black.

Chronic Dysentery.—The tongue is red and glazed, sometimes deeply fissured.

Pyemia.—The tongue is often clean at the outset, but soon becomes glazed and fissured or furred, and after a time dry and brown.

Syphilis.—The presence of patches have already been mentioned.

Bright's Disease.—Appearance similates chronic dyspepsia, which has already been mentioned.

Pneumonia.—The appearance of the tongue is similar to that met with in severe febrile disease, that is, more or less thickly coated with a white fur, and it tends, in severe cases, to become dry and brown.

Diabetes.—The tongue is often peculiarly irritable, red, clean, cracked and dry.

Acute Peritonitis.—It is usually remarkably small and contracted, also red and irritable, with but little fur, and tending to dryness.

Phthisis.—In advanced cases of phthisis, especially with a high temperature, it frequently becomes red and raw, and exhibits enlarged papillæ; the occurrence of thrush upon its surface may also be a sign of approaching dissolution in the disease.

Now I know there is a number of diseases in which I have not mentioned the appearance of the tongue, but the appearances in those diseases are so near alike that I feel it hardly worth your time to bore you longer, especially since they are pretty well covered collectively in the first part of this paper under general conditions and affections of the different organs.

I would like to add a few remarks, taken from an old work on the "Practice of Medicine," published in 1858 by Dr. George B. Wood, and which are not mentioned in any of the later works that I have seen. He gives an extended article on the tongue in disease, under "Symptoms and Signs of Disease," and among other things he says:

"The temperature of the tongue serves as a guide of the body generally. When cold it evinces, for the most part, great prostration of the powers of life. It proves that the process of calorification is failing at the very fountain; for the breath must be cool before the tongue can become so in any considerable degree.

"This coldness of the tongue has been frequently noticed in severe cases of epidemic cholera. But we must take care not to confound coolness from local causes, as from ice in the mouth, or from the patient having slept long with the mouth open in a cold atmosphere, with that proceeding from the state of the system. Heat of the tongue, except arising from inflammation of the organ, may be considered as a sign of a general elevation of temperature.

"The manner in which a furred tongue becomes clean affords valuable indications. When the fur slowly recedes from the tip and edges, thinning gradually as it retires, it intimates a favorable convalescense. A portion of the fur often lingers near the root of the tongue long after the disease has given way. In another mode of cleaning, the fur loosens and separates in flakes, often beginning at the middle or near the root, sometimes in large patches, or over almost the whole tongue at once, leaving a smooth, red, glossy surface, as though the papillary structure had been lost. In such cases, if acute, and if the tongue remains moist, convalescence almost always takes place, though usually tedious, and sometimes very lingering."

I will close my paper by mentioning a few things to remember. Any troubles which cause loss of appetite or retard digestion, such as severe pain, fear, anger, worry, mental overwork, etc., produce a coated tongue. Bear in mind the three clinical aspects which the tongue presents to the practitioner, namely, its sensibility, movements and objective characters.

In my paper I have consulted and taken articles from the following:

"Tongue, Diagnostic Significance of," by Dr. Thomas L. Stedman, in his article on the above subject in Reference Handbook of Medical Sciences.

"The Tongue," by Dr. Frederic Roberts, of London, in his article on this subject in Quain's Dictionary of Medicine.

"Principles and Practice of Medicine," by Dr. Austin Flint.

"System of Medicine," by J. Russell Reynolds, of London.

"Clinical Medicine," by A. Trousseau, of Paris.

"Cyclopedia of the Diseases of Children," edited by Dr. John M. Keating.

"System of Practical Medicine," by American authors. Edited by Drs. Alfred Lee Loomis and William Gilman Thompson.

"A Treatise on the Practice of Medicine," by Dr. George B. Wood and others.—*J. F. Davis, M.D., Oil City, in Pennsylvania Medical Journal.*

---

**This Journal and Atlanta Semi-weekly Journal one year for $1.00 cash in advance. No checks. Strictly to new subscribers.**

### MODERN METHODS OF DISINFECTION.*

Whilst in many spheres of public health, as, for instance, in the removal of refuse, in the building of waterworks, erection of slaughter-houses, etc., health resorts have made satisfactory progress, the disinfection of rooms and clothes has been much neglected; and yet this question is of the greatest importance, for the flourishing of health resorts. It is known to all of us how, through small epidemics of infectious diseases, the importance and extent of which is often exaggerated in the papers, health resorts have been ruined for years, if not forever.

Irrespective of the purely human standpoint, it is also absolutely necessary from the material standpoint that in institutions and health resorts everything possible should be done to prevent the spreading of infectious diseases which have been either introduced from the outside or have originated in the place itself.

Recently especial progress has been made in this respect, and upon which I will now report. It gives me all the more pleasure to do this as, owing to the courtesy of the Actien vorm, E. Schering, I am enabled to practically demonstrate the appliances required for the purpose.

With disinfecting precautions, we must endeavor to hit upon all those things which, according to our experience, receive the germs of the disease of the affected persons, and which could be spread to distant places. Large towns and populous country places possess for this purpose disinfecting establishments fitted up with steam, by which means the germs of disease in the materials are effectually destroyed; in smaller places these establishments are not always to be found. Many articles of use, such as leather goods, decorative articles, etc., can not be disinfected. The important question of room disinfection had to remain for a long time unsolved. Every one who has to deal with this question knows that cleaning of walls and ceilings by means of bread, and washing of floors with antiseptic solutions, even with efficient assistance, give unsatisfactory results. So, for instance, a Swiss author—in spite of the disinfect-

* A paper read by Dr. Hans Aronson, Charlottenburg, at the Eighth Congress of the Allgemeine Deutsche Badeverband of Norderney. Illustrierte Reise-und Bade-Zeitung, 1900.

ing staff having been informed that their work would be checked—has been able to prove that germs, which had been intentionally put into the room, were full of life after the disinfection. From this we can gather what effect would be arrived at if the assistants were less efficient. For a long time we have endeavored to carry out the important disinfection of rooms, together with the materials therein contained, by means of gaseous agents, and it seemed as if our endeavors would prove fruitless, for all gases which were tried—such as chlorine, bromine, and the formerly highly praised sulphurous acid—failed to stand a careful test. Notwithstanding the disinfective action which some of those gases undoubtedly possess (as, for instance, bromine), other conditions remained unfulfilled. A gas must, for the purpose of disinfecting large rooms, possess, for reasons known, almost the same specific gravity as air. Furthermore, it must not be poisonous to human beings. In this respect it must be considered an important advance when formaldehyde was recognized as a gas which fulfilled all those conditions. In my report, published in 1892,* in which I, for the first time and at the same time as Trillat, more closely studied the antiseptic properties of formaldehyde solutions, I had drawn attention to the equal effects of the gaseous form. Many endeavors in the following years pointed to the utilization of this form for disinfecting large rooms. Although this problem seemed very simple, great difficulties were met with in practice. One reason for this was, that our gas very quickly passed into a solid, insoluble, and therefore inactive, modification (paraformaldehyde or trioxymethylene).

I will not tire you with a description of these many experiments, which were continued up to 1897. I then succeeded, by means of Schering's apparatus, originally described by me, in rapidly developing, in an easy and handy manner, sufficient quantities of Formalin gas for the disinfection of rooms. In this now universally known apparatus, the solid polymerized formaldehyde is used for developing active vapors. As early as 1894 † I had demonstrated on many animals and men that this substance is almost non-toxic,

*Berliner Klinische Wochenschr. No. 30, 1892.

†A paper read at the Berliner Verein fur innere Medizin, March 12, 1894. Berliner Klinische Wochenschr., p. 900, 1894.

and was even well borne in large quantities, internally, by children. The utilization of this substance as a developer of formaldehyde gas seemed, therefore, for this reason, to be very favorable. The action of this apparatus consists in this substance—in compressed form of tablets, 15 grs. each—being transformed by means of the hot gases of combustion into the gaseous aldehyde, and these gases of combustion being afterwards mixed with the Formalin vapor. By this means the necessary quantity of moisture is introduced, and polymerization is prevented. By the stream of the gases of combustion, at the same time, a rapid distribution of the active gas in the room is effected. The testing of this method in a large number of experiments gave excellent results, even the most obstinate tubercle bacilli being destroyed with certainty, within twenty-four hours, when using two or three tablets per 100 cubic metre space. Many authors who repeated these experiments (Fairbanks, Grawitz, Kobert) could fully confirm my statements; others obtained less favorable results. It is the merit of Peerenboom, Hammerl, and Kermauner, and, above all, of Flügge, of having pointed out that the air in the rooms to be disinfected must contain a certain amount of moisture so as to make disinfection sure cases in all. Six pints of water should therefore be vaporized per each 100 cubic metre space, either in a kettle, or, simpler still, when using Schering's combined apparatus—Æsculap—which allows the simultaneous development of Formalin and moisture. In this apparatus, the previously mentioned apparatus for gasifying these tablets is surrounded by a ring for the water, which is also heated by the means of spirit. With this apparatus, even authors who had no good results with the ordinary apparatus (for instance, Kurth, of Bremen) obtained very satisfactory results. Another method of simultaneously developing Formalin gas and moisture has been brought out by Flügge. This is the so-called Breslau method, which consists in the vaporization of liquid Formalin, which to a certain extent has been diluted with water. With this method, however, the exact functions of the factors under consideration can not be so easily controlled as with the tablet method. A mistake in measuring the quantity of spirit can, on completed disinfection, be just as little discovered as a mistake in mixing the Formalin, and yet both must be done very accurately in order to guarantee the development of the necessary quantity of active gas. With the combined Æsculap, a glance at the pan suffices to ascertain whether all the tablets employed have been gasified. The certainty of the development of formaldehyde is therefore much greater in this instance, whilst the dosage is easy, handy, and very accurate. The tablets are, contrary to the liquid Formalin, non-corrosive, and, as I said, non-toxic. Although the disinfection by means of tablets is a little dearer, this method shows so many advantages that the cost is not of so much weight, especially if disinfection has not to be carried out very frequently.

Besides these methods, there is another one which has been spoken of, viz., Lingner's glycoformal method, which has been described by Walter and Schlosmann. Here, by means of complicated spray apparatus, a mixture of glycerine and formaldehyde was distributed. This apparatus shows no advantages (provided that by means of the tablets, or Breslau method, equal quantities of Formalin gas are developed), but it has considerable disadvantages. Owing to the difficult construction, the apparatus often fails to act; the gases developed are much more toxic (animals die in rooms so disinfected, whilst with the other method they keep alive), they are exceedingly irritating to the respiratory organs of man, they cling to the articles and walls more closely and permanently, and damage the material itself to a greater extent. There is, therefore, no reason why we should deviate from this approved method of generating Formalin gas and vapour in favour of Lingner's method.

After this theoretical discussion, we come to the important question, how are we to handle the disinfection by means of our gas, in practice, and what may we expect from it? We must not forget that this method, with its valuable properties described, possesses also certain disadvantages. The penetrating power of Formalin gas is limited; it is chiefly a question of superficial disinfection which we can obtain with it.

If materials, such as beds, are soiled on places which we cannot easily reach, as may occur in cases of typhoid, dysentery, or cholera, we must, if possible, have recourse to steam for disinfecting. In diseases such as whooping cough, diphtheria, scarlatina, erysipelas, etc., this is not absolutely necessary, and the bed may be disinfected in and with the room. Care should be taken that everything is properly spread out, and all folds and pockets should be exposed. Flügge has designed special stands for this process. As Formalin does not enter well into small crevices in the floor, washing of the floor with hot soap solution or other disinfecting fluids (sublimate solution) should be carried out, as has previously been the custom, whereby our method is favorably assisted. For practically carrying out disinfection, it is furthermore necessary that the room should be measured, and that all crevices, holes, chimneys, etc., should be closed by means of paper or cotton-wood.

Six pints of water and two or three tablets should be vaporized per 100 cubic metre space. From a table, the number of tablets, quantity of water and spirit may easily be seen for every sized room. When the apparatus is ready the lamp is lighted and the room left. If the room is not required for the next day, I consider it best to let the gas act for twenty-four hours. According to Flügge, eight hours are sufficient for certain disinfection. In special cases the time may be reduced to two or three hours, if six to seven tablets—that is to say, double the above quantity—are used per 100 cubic metres. Disinfection having been completed, the very unpleasant, but absolutely harmless odor of Formalin gas should be rapidly and completely removed. By opening the windows this is only effected after many days.

Complete deodorization may, however, be rapidly carried out by introducing, immediately after disinfection by means of the keyhole, into the still closed room a certain quantity of ammonia. By this means the formaldehyde in the room is transformed into a solid odorless body. From the table already mentioned the quantities of ammonia necessary for every particular case may be seen. The ammonia is vaporized in a special receptacle, heated by spirit outside the room, and the vapour introduced into the room by means of a hole. It is of particular importance to know that all articles in the room are not in any way damaged by the disinfection. Even delicate decorations, furs, leather goods, and books, which cannot stand sterilization by other means, do not suffer by this method. The Formalin disinfection described has splendidly stood all tests in practice. In large towns (Charlottenburg, Breslau), all sorts of dwellings have been disinfected after infectious diseases. Numerous instances in practice have proved that all bacteria coming under consideration have been successfully destroyed.

By utilizing this method to its utmost, and adding a few advantages from other methods, we can now be convinced that room disinfection can be thoroughly carried out—a conviction which no other method up to the present has allowed. I can, therefore, agree with Flügge that we have advanced an important step in the practice of room disinfection, and have for the present obtained all that can be obtained by the means at our disposal. I have pointed out at the beginning of this paper, of what vast importance this question is for the hygiene of health resorts. It is my opinion that the authorities and medical advisers of health resorts and institutions should use this chance of preventing the spread of infectious diseases. I trust that the limit of epidemics will then be drawn with more certainty and more effectually than was hitherto the case.

---

### SHALL WE TELL WOMEN WITH UTERINE CANCER THE NATURE OF THEIR DISEASE?*

No more unpleasant task perhaps ever falls to the lot of the tender-hearted physician, and I think the majority of physicians are tender-hearted, than to have to tell a patient that she is suffering from malignant disease. Some deliberately shirk this unpleasant duty on principle, believing that we are not called upon to tell her the truth in these circumstances; while others avoid it from expediency because they dislike to cause pain, or because they think that it pays better to say something pleasant which will cheer up the disconsolate one for the time and leave a temporary agreeable impression on her mind. In the course of a newspaper

*By A. Lapthorn Smith, B. A., M.D., M.R.C.S., England, Professor Clinical Gynecology in Bishop's College, Montreal.

interview with a great London surgeon, whose name is known all over the world, while speaking of tact as an element of success, he says that the successful physician will not tell a woman that she is suffering from cancer; and one of our greatest Canadian physicians evidently holds the same views, as in consultation over their cases he invariably tells the patient that they will be better soon. As some of these patients were dead two or three weeks later his prognosis was correct in a sense, but not in the one in which the patient would be supposed to take it. The view taken by these two great men, and by many others in the profession, is equally held by a great many of the laity, as seems certain by the number of people who have asked me to tell them truly what was the matter with their sick friend, but on no account to let the sick one herself know the nature of her malady. Not only do the friends almost invariably take this course, but, strange to say, I have known the patient herself beg that I would not tell her the truth.

Notwithstanding all this, I venture to differ from these doctors, from the patients and their friends, and I wish to enter a firm protest against any other course being adopted than to tell the truth every time; and I think I can show that this is our duty for many reasons. For those (and I hope they are few) who require any other argument than duty, I think that I can prove that it does not pay to deceive a patient even under the mistaken idea that good may come of it.

I am not saying anything about telling the friends what is the matter; for when they have brought me a patient and mysteriously called me to one side to whisper that I could tell them, but not to tell the patient, I have often said to them, "the patient is the only one who has any right to know; what is the matter with her and what she tells me are alike profound secrets; I will tell her and she can tell you if she likes; but I will not tell any one else."

Is it right to tell the truth? At first sight I will admit that it appears more humane not to tell a woman that she has cancer of the womb, but in reality this course is most cruel; for, in each of the three stages into which I will divide the progress of the disease, the woman has an immense stake at risk; she has everything to lose and nothing to gain by being kept in ignorance of her actual condition, and she has a right to know it whether she wants to or not, from the moment the physician knows it himself. In the first stage of cancer of the womb the disease is absolutely local; that is to say it occupies a small spot on the angle of a laceration or is limited to the endometrium. During this stage the disease is positively and entirely curable by the removal of the womb. Vaginal hysterectomy for cancer in this stage by a good operator has at the present time a death rate of two or three per cent. at the very most. Alas, it is bad enough to think that so few cases are even diagnosed at this stage, without wishing that the few which are diagnosed should be deceived into a fool's paradise, while their physician, whom they trust to save them when they could be saved, quietly stands by and with the friends becomes a conspirator to hide their danger from them, until it is too late to escape it. How can she hold out her hand for help if she does not know that she is in danger. I for one decline to place such a responsibility upon my conscience or to become a plotter against her life, as I would be if I knowingly and willfully concealed from her the one fact that she had cancer of the womb, and the other that vaginal hysterectomy would save her. I shall never forget the remorse of a medical friend who brought me a patient with a large cancer of the uterus with the broad ligaments infiltrated with the disease which he had been treating with caustics for six months, and which was then too far advanced to permit of hysterectomy; looking me straight in the eyes he asked me whether, if he had brought her when he first discovered it, I could have saved her, and I was obliged to answer that I believed I could have done so. He only replied, "I will never forgive myself," and from the tone in which he said it I believed him. And yet he only lost her life through unintentional ignorance of her true condition and not through willful deceit, and he was not therefore anything like so much to blame.

In what I call the second stage the cervix is invaded, the broad ligaments are full of cancerous tissue and the lymphatics are infiltrated with it. The patient is alternately being drained of her life blood by irregular hemorrhages, which she attributes to the change of life, and poisoned by the absorption of decomposing and gangrenous material, which keep her in a burning fever. Her presence, moreover, is made loathsome to her friends by reason of the foul smelling discharge. And yet she does not know that she is a hopeless case of cancer of the womb. Why tell her? Because we can prolong her life, diminish her suffering, and not only thus make her life endurable to herself but render her unoffensive to her family by a thorough use of the curette and cautery. Under an anesthetic we can do all this without any danger or causing any pain, and in a few days she will feel so well that she almost doubts the accuracy of the diagnosis which she has to thank for her improved condition. I have had, unfortunately, too many of these cases and two few of those in the first stage, but I have had the pleasure of seeing apparently dying women almost at once become comparatively well. In a few cases I have curetted them a second time at the end of three months more, but as a rule the one curetting has sufficed, and they have died peacefully and painlessly from cancer of the liver from six to nine months later, surrounded by their friends to whom they were no longer an object of disgust. Now, how can they avail themselves of this merciful treatment unless they know the necessity for it? It is more than we can expect, and certainly more than we often find, a woman willing to submit to an operation without knowing the nature of the operation and why it is to be done. If their condition is made known to them they readily consent to have it performed.

In the third stage, where the whole pelvis is filled with a cancerous mass and the bladder and rectum involved, and nothing is to be done except to make death painless, what should be our answer to the woman who asks us what is the nature of her disease, and whether we can cure her. I have had such patients ask me if they could be quite cured in a month, when as events turned out they were fated to be dead and buried in less than that time. Shall we follow the golden rule and do to this poor woman as we would be done by if our positions were reversed? And would we not wish to know the truth so that we might prepare for death?

At the beginning of this article I said duty should be our first consideration, and I hope I have shown that it is our duty in this case, as in all cases, to tell the honest truth. But even from the infinitely lower standpoint of expediency, I hope to show that it pays to tell the truth.

When we hear a friend lying from kindness or expediency, does our opinion of that friend go up or down? When we have heard him telling a woman with cancer that she will be all right soon, and telling us five minutes later outside the house that he believes she will be dead in a week, and when we have heard him saying the same two opposite things in twenty other cases, will our faith in his word be as implicit as it would be if we had heard him on every occasion telling the truth, the whole truth, and nothing but the truth? If we weakly comply with the request of the friends and lie to a patient, what will these same friends think of our truthfulness when it is their turn to consult us? Even if we told them the truth, would they believe us? Certainly not, if they knew that we had lied to others in the same circumstances.

We see the effect of this mistaken policy almost every day in patients who have been under several doctors. When we go to examine them they ask us if we will hurt them; if we say "no," they exclaim, "I know you will." If we are going to hurt them it is better to tell them so beforehand, and they will bear it quite bravely. It is only after the second or third visit that we will succeed in winning their confidence by telling them the truth every time; and then when we pick up the speculum they no longer jump up saying, "Is that a knife you have in your hand?" By showing them each instrument and explaining what we are going to do, they will no longer fear or doubt us. And this is how it pays to tell the truth; little by little you will build up a circle of friends who will tell their friends that they can depend upon what Dr. so-and-so tells them, and the staunchest friend of all will be the poor dying woman whom you have given a chance to prepare for death. Next to her will be the woman who has been curetted and made comfortable and endurable to her friends; and last but not least the woman will thank you for the truthfulness to which she owes her life, and by which she has been saved from a painful and loathsome death.

Not only does the reputation of, and faith in, the individual physician increase in proportion as his word can be trusted implicitly, but the faith of the public in the profession as a whole would be still greater if even in these distressing cases, no matter how good and kind the motive may be, our invariable rule with every one of us would be to leave nothing undone to make an early and accurate diagnosis, and once that has been done, not to lose a single hour in having the proper treatment carried on.—*Can. Med. Record.*

---

### SYPHILIS AMONG ALASKAN INDIANS.*

Having had occasion recently to visit several Indian villages in southeastern Alaska, I had the opportunity of observing interesting cases of different kinds but I will confine myself in this paper to the subject of syphilis among the natives of that region.

As a rule the Indians do not care for any other than their own "medicine man," and it was only because of having been called to treat the results of a cutting affray which their own "tom-toms" failed to cure that I obtained the entree into Killisnoo society. The original rumpus was caused by the drinking of "Pain-Killer," a tipple that I have not seen on the ordinary American bar. The Indians visit every government vessel and beg for medicine, they generally get bicarbonate of soda. I believe it is simply because they want something for nothing, as they are perfectly indifferent as to what they get or what may be the effect.

My opportunities for studying cases thoroughly were limited, for the vessel I was on was constantly on the move; no histological work could be done, for we had no microscope and the natives are so mortally afraid of a scalpel that I could not procure any specimens. Ten years ago I was in the far north on the Siberian coast and in the Arctic, and although at that time I had no medical experience the pictures that I saw on the bodies of the Esquimaux have never faded from my mind. Ever since then I have been bewildered during my work in the University of California clinics that such pictures did not repeat themselves among the great number of syphilitics that came for treatment. This last visit to

*Read by Wm. Gilbert Hay, before the California Academy of Music.

Alaska has opened my eyes. The disease exhibits itself in an exceptional fashion among these people. As a partial explanation, the hygienic condition of their houses is abominable. Civilization has taught them to build the semblance of an American house and the value of a dollar, but that is all. The house contains one room in which they perform all domestic duties—five or more inmates in the room; all the windows are nailed down, and a fire is going nearly all of the time, mostly in a small American stove, and needless to say the atmosphere is stifling. There they squat until it becomes imperative to go fishing or to do other mild work and they wobble out into the cool air, though not very cold without additional clothing, and the children go bare foot. Many have rheumatism. Why they all have not passes my comprehension. In the second place the Indians take no real treatment for their diseases, contrary to the story books that tell us about marvelous herbs.

Case I. Indian Klootchman, lues, ten or more years duration; never had treatment. Bridge of nose sunken; three scars from old gummata on forehead; buccal cavity and adnexa intact; general adenopathy; both legs and thighs greatly deformed from previous gummata and covered with reniform ulcers size of a palm; ulcers circular, when not reniform, dirty bases, high rolled, indolent edges; left buttock presents four large spreading ulcers; shagreened and scaling eruption on dorsum of right foot; scales round and ¼ inches in diameter; slight desquamation; nothing on upper extremities; palms, nails, hair and teeth good; native dressing of chewed cedar bark with some red clay on ulcers; had not left the house for seven years. Treatment: hydrogen, peroxid, ung. hydrag. kal. iodid, 10 grains t. i. d.

Sept. 26, repeat treatment: All ulcers healed except one on buttocks. Except for the extent of the disease this case differs in no respect from those seen in this city, but there are others that give food for thought.

Case II. Native, aet., 30 years. Suppurating, shallow cavities, probably broken down gummata on lower and posterior portion of scrotum—in fact the perineum. No superficial scarring, but an inverted edge resembling the retracted nipple in scirrhus of the breast; sinuses leading toward the left testicle, but do not seem to connect; left testicle very large and hard; right testicle apparently normal; universal adenopathy; present condition has existed for seven years, according to patient's narrative; no previous treatment; history of some skin eruption "years ago."

Case III. Indian about 50 years of age. Similar ailment as preceding case, but both testicles were involved, large and hard, and the sinuses more numerous. Reniform ulcers on the knees both bends of the elbows, anterior surface, and inner sides of both thighs adjoining the crotch. Distribution of ulcers symmetrical.

One objection will be made: Was my diagnosis correct? It is well known that fistulas and ulcerations with involvement of the skin of the scrotum is rare in syphilis while common in encephaloid carcinoma and tuberculous orchitis. But there was no effusion, no large veins over the scrotum, tumor hard and involving both testicles. Further other evidences of lues were present and the therapeutical test was successful.

Incidentally a number of doctors connected with different Government departments as well as casual visitors have said that there is much tuberculosis among the natives. All that I care to say at the present time is that their education has been neglected. One instance will amuse you. At the Presbyterian Mission in Sitka a homeopathic physician showed me a native woman whose entire arm he intended to amputate for a slight luetic periostitis of the fore-arm. She had never taken anti-syphilitic treatment. He was very courteous and I dislike to repeat this, but he was to be assisted by a regular physician from one of the Government vessels who, however, I am pleased to say, refused on second thought and some advice. Referring to the two cases last cited, I believe that Unna's classification of such syphilides is correct; something of which I had not been convinced before. They are late tuberous syphilides and not gummata proper.

I will attempt to sum up my observations in Alaska as follows: In about forty cases of lues I did not see one in the condylomatous stage. I did not see a single affection of the mucous membrane. No perforated palates. Eight cases of specific iritis, one with synechia, one completely blind. Many eye cases were reported to me by the Russian storekeeper. Every Indian examined for any trouble whatever had a general adenopathy, including the epitrochlear glands, for which I particularly looked on account of the statement once made that their enlargement is pathognomonic of syphilis. This observation holds true of the sailors, some hundred of whom I examined. There was in no case any affection of hair, teeth or nails.

---

### OFFICE DISPENSING.

There have been advanced pro and con many arguments relative to the desirability of the physician dispensing his own medicines. The nature of these has been varied, vascillating between those of a purely business and pecuniary bearing on the one hand, and those of an ethical tincture on the other. The question is of such a perennial and abiding nature that most medical men have been obliged to practically settle it for themselves in one way or another, and for reasons that seem to them competent and expedient if not wholly decisive from the respect of a general principle. A spirit of self-defense has served as the chief factor in the conclusion of some, while others have been equally guided in their action by a sense of responsibility, in assuming the duties of dispensers as well as prescribers. It can readily be understood that, under circumstances of jealous rivalry, or combinations of competing effort or inefficiency of one kind or other, a conscientious practitioner even otherwise disposed might hesitate to trust the welfare of his patient and himself to any other than his own supervision. Especially is this likely to be the case in small towns and villages where there is little or no opportunity for choice. Where there is no apothecary or druggist, the question necessarily resolves itself into one of personal necessity. Then again, there are those who consider it a matter of education for themselves, and a convenience if not a duty to their patrons to be able to at once and without loss of time adjust the remedial agents to the needs of the case. The former of these positions is unquestionably a tenable one, and the latter has much in favor of its justice and convenience. In the former instance it may be argued that the individual who manipulates his own weapons must come to know them better and handle them to better advantage. He is more likely to change or vary them oftener when required, and to watch their action more closely and become more familiar with the varying shades of influence following change in dosage and combination. There can be no question, in the opinion of the writer, that this is true, and that much that is now left to the laboratory experimenter and clinical observer in the more concrete dispensary centres, was more effectively accomplished in times past by the men who themselves prepared and closely watched the action of essences from the roots and herbs of the forest and field. As to the desirability of this habit from the second point of view, it is only necessary to point to the degree of confidence and satisfaction evinced by the patient and friends where the physician proceeds to at once dispense what he wishes in the case, and to the unanimity with which that feature of homeopathic and kindred practices is publicly indorsed.

But aside from the classic arguments reiterated in the foregoing brief resume, there is one of more recent development and growing import that is bound to have its weight in determining the future status of office or personal dispensing. That is the tendency of the patient, if not the druggist, to usurp the prerogative of the physician and re-prescribe for him or herself. This is without doubt becoming one of the greatest evils of the time and one before which the dangers of the use of patent medicines sinks into insignifiance. In consequence of the general intelligence, perhaps, or the curiosity or reading habit of the laity, a prescription is seldom written that cannot be and is not at once read by the patient and it contents noted. In this way he readily familiarizes himself with the nature of what he is taking, and the result is that if he feels the better for it he is more than likely to continue its use, or possibly abuse, oblivious or careless as to remote consequences. In this way doubtless is often inaugurated the opium, chloral, cocoaine and other reprehensible and debauching drug habits. As a matter of fact the welfare of both patient and physician dictate the necessity for keeping the subject in absolute ignorance of what he is taking. In no other way can these prevalent evils of which we speak be held in subjection, and seemingly in no other way can that be accomplished than by the physician himself furnishing to the patient just what he desires him to have, and when, and insisting upon it as his right and duty to dictate the terms of its administration at all times and under all circumstances. This, to our way of thinking, is one of the strongest arguments that can be advanced in favor of the physician dispensing his own medicine, and keeping the patient and all else in absolute ignorance of the nature of the influences under which he is placed. We have long been favorable to the system of office dispensing, and the more we see of the growing tendencies of the time the more impressed are we with the necessity for some of the safeguards which it undoubtedly affords. The only real effective objections to the habit are those based upon the economy of the practitioner's time, and the more important matter of the protection which is sometimes afforded by the corrective knowledge of the pharmacist, and the availability of a written record in the event of malpractice claims. While of undoubtedly great weight, these objections seem not as insuperable as are the detailed evils which accompany and follow the indiscriminate habit of prescription circulation and self-medication that at present prevails.—*Peoria Medical Journal.*

---

### EPIGAEA FOR ERUCTATION.*

I wish to suggest epigaea as a remedy for belching up of tasteless or offensive gases.

Epigaea repens, commonly known as trailing arbutus, ground laurel, gravel plant, and in New England as mayflower, is a North American plant and grows throughout the belt which extends from the Mississippi eastward to the coast north of the Ohio river. It grows generally upon rich, damp, mossy banks under the shade and protection of the low pines and hemlocks which abound in this region, and blossoms during the first few sunny days of spring amid the verdure of the previous year's growth.

Owing to the time of its appearance, the beauty and fragrance

* Read by Charles D. Aaron, M.D., Detroit, Mich., at the annual meeting of the American Gastro-Enterological Association at Washington, May 1, 1900, and contributed exclusively to the *American Therapist.*

of its flowers, trailing arbutus is one of the most widely sought of all the spring flowers of the North Central States.

Epigaea repens may be described as a phenogamous, monopetalous exogen, a member of the heath family proper, and as such closely related to gaultheria procumbens and uva ursa.

Its roots are fibrous, perrennial, reddish brown in color, massed together with many rootlets. The stem is roundish and conspicuously hairy; the bark also has hairs and is rusty brown in color. The leaves are alternate, evergreen, reticulate, ovate-cordate and entire, from one to two inches long and relatively half as wide, the edges and under surface rusty and hairy. The inflorescence is axillary, the flowers spring from dry, scaly bracts, are deep rose, delicate pink and sometimes even white in color, emitting a fragrant spicy aroma. The flowers have sepals, dry and nearly separate, corolla deeply parted, ovate spreading lobes, the tube hairy inside, ten stamens, shorter than the corolla, filaments heavy at the base, anthers linear, opening longitudinally, ovary globular, five celled, many seeded, style slender, forming a zone about the minutely five lobed stigma.

Although the plant has not been recognized officially, it has received some attention at the hands of the medical profession. With regard to its active principles, three glucosides have been isolated —arbutin, ericolin aud urson. Formic acid and a body similar in many respects to gallic acid, tannic acid to the extent of three and one-half per cent., and ericinol, a pale, yellow, aromatic oil have been accredited to this drug. According to Thal, ericolin appears in five species of ericineæ, and all of the three glucosides, in fact, have been proven identical by Mr. Oxley (1871) with the same glucosides found in uva ursa. Its medical uses are, therefore, similar in many respects to those of uva ursa, and like it can be used freely without fear of toxic symptoms.

I have had many happy results with it in some very obstinate cases of eructation of gas. I am unable to say just how the drug acts. Its results, however, are seen almost immediately. I prescribe usually one dram of the fluid extract to be given three or more times a day after meals. On account of its principles being complex, it is impossible to state to which one of them the therapeutic value is due.

If the eructation be due to fermentation it may be that the aromatic oil acts an anti-ferment, yet results are equally seen in nervous eructation or in cases when the patient belches up air that he has swallowed. It seems to act well in all cases of eructation dependent upon neuropathic conditions.

I simply wish to suggest a resort to this drug when others have failed, for I feel there are times when we are able to stop some cases of persistent belching by means of it.

The pharmacogical data have been compiled by Mr. L. A. Seltzer, of Detroit.

The following references are suggested: Shoemaker, Materia Medica; Johnson, Medical Botany of North America; Gray's Field Book; Bessey, Botany; Proc. Amer. Pharm. Asso., Vol. XXXII., pp. 147; Pharm. Zeit. Russel., 1883, Nos. 14–18.—*American Therapist.*

---

### UNNA'S DRESSING FOR CHRONIC ULCER OF THE LEG.

R. S. Michel, in the Chicago *Clinic* for August, strongly recommends Unna's dressing in the treatment of chronic ulcers of the leg.

It is prepared thus: Take of water and glycerine, each ten parts, and of gelatine, and white oxide of zinc, finely powdered, each four parts. The gelatine should be of the best quality. Dissolve the gelatine in the water by means of a water-bath. While hot add the glycerine, and finally the oxide of zinc, stirring vigorously and continuously until cold. It is somewhat difficult at first to prepare it well, but with a little experience the art of doing so is readily acquired.

Prepare the leg by washing it thoroughly with soap and water. Carefully dry it, and rub it with alcohol. Then the "paint," prepared as above, and having previously been melted, is to be applied to the leg with a paint brush, just as you would apply paint. Paint over the ulcer, paying no attention to it. Leave out the toes. Extend dressing nearly to knee. Keep the paint hot while using it by putting the vessel in which it is contained into another vessel containing hot water—an extemporized water bath. Stir from time to time, in order that the mixture may be homogeneous. After the leg has been thoroughly painted, apply an ordinary bandage. The bandage should be two or two and a half inches wide and made of firm but course material, with open meshes. The painted surface must be evenly and accurately covered, but the bandage must make no wrinkles or reverses. This makes it necessary to put it on in pieces. Let the bandage take what course it will, and when it cannot be applied further without making a wrinkle, cut it off and start anew. Keep on in this way until the whole painted surface is covered with the bandage, being careful to close in the heel and ankle joint well. Apply to this bandage another coat of paint, just as was done in the first place to the bare leg. Then apply another layer of bandage, fortiyfying any weak spot with an extra piece of bandage, and more paint, so that there is exerted an even pressure all over. Then another coating of paint and bandage. The dressing should be from three to five bandages in thickness. Complete the dressing by giving the last bandage a good coating of paint. For a few hours the completed dressing is somewhat sticky; so it is well, for the sake of cleanliness, to cover it with an ordinary roller, which may be removed later.

The essential points to be remembered in applying the dressing well are: to make it fit firmly and evenly all over; it should fit like a glove; do not try to put on too long a strip; use plenty of paint; leave no weak spots.

The dressing should wear from four to eight weeks. Should it become loose from a diminution in the size of the limb, remove it and apply another. As ulcers of the leg are usually accompanied by a swollen or œdematous condition, and as this condition rapidly diminishes under this treatment, the dressing may need frequent change. Should the ulcer secrete pus, rendering the use of local applications desirable, a wet spot will make its appearance on the dressing over the seat of the ulcer, within a few days after its application. In this case a fenestrum may be cut through the dressing sufficiently large to expose the sore. Through this hole the necessary application can be made, and the fenestrum closed evenly and nicely with cotton, or gauze, held in place by means of a bandage. The application may be europhen, iodoform, boracic acid, or whatever cicatrizant is preferred. As the ulcer rapidly become smaller it is well to make the fenestrum only large enough to expose it.

This plan of treatment is applicable to all ulcers of the leg, from whatever cause they may be produced. In some cases in which there is *great varicosity,* ligation and excision should be done first. If the ulcer is syphilitic, the constitutional treatment should be conjoined.

It is also applicable in many cases of chronic eczema, and to cases os œdema from whatever cause. In cases of synovitis, or chronic sprain, in either the upper or the lower extremity, it can be used to secure fixation of the affected part. This it accomplishes nearly as efficaciously as, and much more agreeably than, plaster paris.

The dressing can be made to answer other indications than those enumerated.

We trust our medical friends will give this plan of treatment a trial.—*Charlotte Medical Journal.*

---

### FORMALIN AS AN ANTISEPTIC IN GENERAL SURGERY, GYNECOLOGY AND OBSTETRICS.

G. E. Crawford, Cedar Rapids, Ia., is convinced from his own experience that formalin, the 40-per-cent. solution of formaldehyd, comes nearer meeting all the requirements of a perfect disinfectant than any other substance yet employed. Its germicidal potency ranks with that of the two or three most efficient bactericides, and its inhibiting power is probably greater and more lasting than that of any of them. It is not only an antiseptic, but a deodorizer as well, and solutions of potent strength are much less irritating, both to the hands and to wounds, than any other equally efficient antiseptic. It is not injurious to instruments and it is practically innocuous. Large cavities can be freely irrigated, even the peritoneal cavity, and a considerable quantity of the solution can be allowed to remain without injury or danger of toxic effect. The solutions that have generally been recommended are too strong. The strength of a solution depends upon the amount of formaldehyd it contains, and it should be remembered that this is two and a half times stronger than formalin. The latter is a definite substance, and is the tangible form in which we employ formaldehyd as an antiseptic in surgery; therefore the percentage of a given solution should be that of formalin and not that of formaldehyd. The solutions of formalin commonly used are the eighth, fourth, and the half per cent.; the two former for washing infected wounds and irrigating and packing cavities and sinuses, and the latter for disinfecting the hands and the surface of the body. They contain formaldehyd in the strength of one two-thousandth, one one-thousandth, and one five-hundredth respectively. The half per cent. solution is of about the right strength for the hands. In gynecologic practice, formalin is extremely useful; the peritoneal cavity can be freely irrigated with the one two-thousandth solution in cases of pelvis abscess, pyosalpinx, etc., where purulent matter has escaped into the cavity. It is superior to iodoform in packing and drainage of sinuses, while in obstetric practice it meets all the requirements of an antiseptic to disinfect the hands and the external parts.—*N. Y. Med. Jour.*, *St. Louis Med. Review.*

---

### DIGITALIS IN THE TREATMENT OF HEART DISEASE.

Dr. W. H. Washburn, in *Merck's Archives*, says: "The action of digitilis is upon muscular fiber, causing it to relax more slowly and to contract more quickly and perfectly. Its effects are not alone limited to the heart, but extend to the muscular system in general. Digitalis increases blood pressure and converts the intermittent circulation in the capillaries into a steady flow. The increased blood pressure lessens the rapidity of the heart's action, as well as does the action of the drug upon the vagus. The whole amount of blood in the arterial system is increased, and there is a corresponding lessening of the amount in the veins. This stimulates the absorption of serous exudates. The myocardium, like other tissues of the body, receives an increased blood supply, and consequently its nutrition improves, thus favoring compensating hypertrophy. Digitalis is not contraindicated in aortic regurgitation and full pulse. The administration of digitalis is much hampered by fear of its cumulative effects. This is a real danger, as it is a drug that is absorbed slowly, and elimination is by no means rapid. In consequence we may have a chronic or acute intoxication if the doses are small and administered at short intervals. Chronic poisoning by digitalis results in contracted arterioles and in increase of resistance in the peripheral circulation. The coronary arteries, in common with other arterial trunks, share in this condition, and consequently, as a result of too free administration of the drug, the nutrition of the myocardium is lessened, with a consequent aggravation of the heart failure.

The dose of digitalis that will prove tonic to the heart is a moderate one; this dose must only be repeated after a sufficient interval. If the dose be excessive and the intervals between the doses too short, unfavorable results may follow. A fair dose of general application is the use of one grain of the powdered leaves, or its equivalent in any of the official preparations, administered once in twelve hours. This is a dose that can be continued for months or years without saturating the system. Such doses may not produce the immediate results which are often desired by the friends of patients, but under its administration a feeble, almost imperceptible pulse gradually improves, with a corresponding amelioration of the general symptoms. If large doses are employed, symptoms of saturation should be looked for, which is shown by a slowing of the pulse-rate, lessened diuresis, and more rarely diarrhea.—*Indian Lancet.*

---

### HYDROPHOBIA INSTITUTE ELECTS OFFICERS.

The organization of the Georgia Pasteur Institute and Laboratory has been perfected. The committee and charter members of the institute met in Dr. C. D. Hurt's office Thursday evening and elected the following Board of Governors:

Dr. C. D. Hurt, Dr. James N. Brawner, Dr. Claude A. Smith, George C. Waters, of Atlanta; Dr. Henry R. Slack, of LaGrange; Dr. J. H. McDuffie, of Columbus; Dr. St. J. B. Graham, of Savannah; Benjamin W. Hunt, of Eatonton, and Dr. E. P. Ham, of Gainesville.

The Board of Governors then elected the following officers:

President, Dr. Henry R. Slack, of LaGrange; vice-president, Dr. J. H. McDuffie, of Columbus; treasurer, Dr. C. D. Hurt, of Atlanta; secretary, Dr. Claude A. Smith, of Atlanta.

Committees were appointed to solicit subscriptions to stock and also to select a site for the institute and laboratory. Everything points to an early opening of the institution.

The Georgia Pasteur Institute aud Laboratory is expected to prove of great importance both to the State and the South, as there are now only three such institutions in the United States, and none nearer than Baltimore. One of the features of the institute will be the treatment of hydrophobia by Pasteur methods.

There have been five deaths of hydrophobia in Georgia among whites that have been reported, and it is estimated that fully an equal number of cases have not been made public.

---

### THE MODERN TREATMENT OF DIABETES MELLITUS (DR. R. SAUNDBY).

After reviewing the modern theories of the causation of diabetes, and excluding the transitory cases, the writer classifies all cases as being either of nervous, pancreatic, or hepatie origin. He defines diabetes as a persistent or recurrent glycosuria which sooner or later is accompanied by thirst, polyuria, and failure of nutrition. The first step in the treatment of this disease should be to ascertain the effect of withdrawing all carbohydrate food, in order to determine how much carbohydrate food the patient still retains the power to assimilate. The writer gives the number of heat units (in food) that a man needs during rest and exertion, and also an example of an average non-carbohydrate diet for a patient requiring 2,400 heat units a day. The greatest difficulty arises from the need to deny diabetic patients both bread and potatoes. Six ounces of potatoes are enough for one meal, and would enable the patient to dispense altogether with any bread substitutes. Bread is best given in the form of toast, but as an ounce at a meal is as little as a patient can do with, and as this is equal to 1,000 grains of sugar a day, he is better without it. Alcohol should be given in the form of Scotch whisky or light wines. Where the patient seems to handle small amounts of carbohydrates with safety, certain "extras" may be permitted. Examples of diets containing these extras are given, and cases are reported showing the results of their use. Finally, the writer gives a very useful table showing the carbohydrates or sugar-forming substances contained in various articles of diet which may be used in limited quantities by diabetic patients.—*Lancet.*

---

### TARNIER'S MONUMENT.

The monument to the memory of Dr. Tarnier has caused considerable discussion among members of the hospital corps of Paris. The monument is a high relief in marble, and will be placed on the wall of the hospital where the celebrated surgeon held his clinics. The artist had represented the doctor as operating upon a patient surrounded by a number of assistants that were portraits of well-known specialists, hence the trouble. The dean, Dr. Brouardel, settled the contention by having the model changed so that Dr. Tarnier is assisted by three students whose features are not portraits. A scheme for perpetuating the memory of living specialists was thus defeated.—*Ex.*

---

### AN AFRICAN REMEDY FOR DYSENTERY.

The Kafirs and Zulus make use of the root of the geranium, of which there is said to be a number of varieties, all, however, of equal therapeutic efficacy, in South Africa, in the treatment of dysentery. They simply chew the root, but the British army surgeons give it in the form of a decoction in milk. The remedy is reported by those who have employed it to be a real specific, no failure to cure within thirty-six or forty-eight hours being recorded.—*Medical Record.*

